# Experiments in no-impact control of dingoes: comment on Allen *et al.* 2013

**DOI:** 10.1186/1742-9994-11-17

**Published:** 2014-02-22

**Authors:** Christopher N Johnson, Mathew S Crowther, Chris R Dickman, Michael I Letnic, Thomas M Newsome, Dale G Nimmo, Euan G Ritchie, Arian D Wallach

**Affiliations:** 1 School of Biological Sciences, University of Tasmania, Private Bag 55, Hobart, Tasmania 7001, Australia; 2 School of Biological Sciences, Heydon-Laurence Building A08 University of Sydney, Sydney NSW 2006 Australia; 3 Australian Wetlands and Rivers Centre, School of Biological Earth and Environmental Sciences University of New South Wales, Sydney NSW 2052 Australia; 4 Department of Forest Ecosystems and Society, Oregon State University, 321 Richardson Hall, Corvallis, OR 97331, USA; 5 Centre for Integrative Ecology and School of Life and Environmental Sciences Deakin University, 221 Burwood Highway, Burwood, Melbourne, Victoria 3125, Australia; 6 School of Marine and Tropical Biology James Cook University, Townsville, Qld, Australia

**Keywords:** Mesopredator release, Trophic cascade, Red fox, Feral cat, *Canis dingo*

## Abstract

There has been much recent debate in Australia over whether lethal control of dingoes incurs environmental costs, particularly by allowing increase of populations of mesopredators such as red foxes and feral cats. Allen *et al.* (2013) claim to show in their recent study that suppression of dingo activity by poison baiting does not lead to mesopredator release, because mesopredators are also suppressed by poisoning. We show that this claim is not supported by the data and analysis reported in Allen *et al.*’s paper.

## Introduction

One of the most vexed issues in the management of Australian wildlife is how to protect livestock from predation by dingoes (*Canis dingo* Meyer 1793). The usual approach to this problem is to attempt to suppress dingo populations by distributing meat baits laced with poison [1]. There are two problems with this. First, such attempts at lethal control of dingoes are often ineffective, and may even result in higher stock losses if surviving or recolonising dingoes change the way they interact with livestock [2]. Second, if poisoning does succeed in reducing dingo populations it could allow increases in the abundance or activity of other species normally controlled by dingoes, with cascading impacts on livestock production and biodiversity. These other species might be herbivores with potential to damage habitat and compete with livestock if over-abundant [3,4], or mesopredators including the European red fox (*Vulpes vulpes*) and feral cat (*Felis catus*) [5,6], which threaten small and medium-sized vertebrates in many Australian environments [7].

In a recent paper, Allen *et al.*[8] report the results of experiments on the effects of lethal control of dingoes. They worked on six cattle stations in northern South Australia and Queensland, selecting a large section on each property as a treatment area while another area on the same property with a similar baiting history was designated as a control. Activity of dingoes and three mesopredators (red fox, feral cat and goanna *Varanus* sp.) was measured on each area (throughout, ‘activity’ is represented by counts of tracks on sand plots). Then, they applied a differential poison-baiting regime in which the treatment area was baited twice per year for two to three years, and the control area was left unbaited.

Allen *et al.* make two claims from the results of this experiment: (i) as a result of poison-baiting, activity of dingoes was “demonstrably less” in baited areas; and (ii) increase of mesopredators did not occur in response to these reductions because those smaller predators were also suppressed by baiting, with the result that mesopredators were in “similar or greater abundance in unbaited areas relative to baited areas”. Allen *et al.* conclude that poison baiting of dingoes does not lead to mesopredator release. The purpose of this comment is to make it clear that this conclusion is not supported by Allen *et al.*’s data.

### Did baiting reduce dingo activity?

Allen *et al.* tested for an effect of their baiting programs on dingo activity by averaging all estimates of activity through time and contrasting mean values on their treatment and control areas. They found significantly higher dingo activity in unbaited areas at four of the six experimental sites. On the face of it, this suggests that baiting tended to produce sustained reductions in dingo activity. However, at two of these sites (Mt Owen and Quinyambie), the activity of dingoes was already higher on the ‘unbaited’ areas before the differential baiting treatment was applied (see Allen *et al.*’s Table two and Figure two). These differences did not obviously change thereafter, and so cannot be attributed to baiting. Presumably, they were caused by other differences between those areas that were not considered in the analysis. These two sites happened to be the ones with the highest measures of dingo activity on the sites that were allocated to the ‘unbaited’ treatment, so they make a large contribution to the difference in mean activity on ‘baited’ *versus* ‘unbaited’ sites.

This observation points to a serious problem in Allen *et al.*’s analysis of their data. Their experimental design should have allowed them to compare the difference in dingo activity on paired study areas before and after the imposition of the differential baiting treatment, and so attribute changes in mean activity to the effect of their baiting programs. But they did not make this before/after comparison: they compared activity on baited and unbaited sites after taking means of all measurements of activity, pooling samples from before and after application of their treatment. It remains unclear whether baiting did cause sustained reductions in dingo activity on their sites. We requested Allen *et al.*’s raw data so that we could check for such an effect, but they were unwilling to provide it. Inspection of Allen *et al.’*s plotted data (their Figure two) suggests that the effect of their baiting programs on mean dingo activity was in fact weak or absent in most cases.

In a separate analysis, Allen *et al*. tested whether individual episodes of bating caused short-term reductions in dingo activity. They did this by comparing activity of dingoes before and soon after individual episodes of baiting, and making the same comparison on paired unbaited areas surveyed at the same times. They conclude that baiting produced rapid reductions in dingo activity, but that dingo activity then recovered towards pre-baiting levels, presumably due to immigration. However, their own analysis of their data refutes this. A short-term effect of baiting would be revealed by a significant time-by-treatment interaction in the ANOVA that Allen *et al.* used to analyse these data. The interaction term in their ANOVA was weak and non-significant, showing that knockdowns due to baiting were either too small or too transient to be distinguished from background variation in activity.

We note in passing a third problem with Allen *et al.*’s analysis. Their data on activity of dingoes and other predators are highly skewed and contain many zero values. Data with such skewed distributions ought not be analysed by conventional parametric methods such as ANOVA unless normalised by appropriate data transformations. This was evidently not done. For this reason alone, all inferences that Allen *et al*. draw from their results should be regarded as unreliable.

### Did baiting suppress mesopredators?

So, Allen *et al.* have little support for their inference that baiting affected dingo activity. There is even less support for their conclusion that smaller predators were affected by baiting, such that they showed higher activity on unbaited areas relative to baited areas. The problems here are similar to those detailed above. At only two of the six experimental sites (Cordillo and Quinyambie) was average fox activity significantly higher on unbaited than baited areas, but these differences were present before the differential baiting treatment was applied and so cannot be attributed to the effects of baiting. There are no indications of differences in activity with respect to baiting for feral cats and goannas at any of the study sites. The analysis testing for short-term responses also found no effects for foxes, cats or goannas.

The lack of evidence for increased fox activity in unbaited areas should have been surprising to Allen *et al.* Foxes readily take poison baits laid for dingoes and, other things being equal, should have increased their activity in the absence of baiting. Instead, one of the strongest patterns in Allen *et al.*’s data is that fox activity was consistently low at all of their sites, and at almost all times. Activity indices for foxes were typically about 5-10 % of those measured for dingoes, and many surveys returned zero values for fox activity. Other research using similar methods in the same region has shown that measures of fox activity varied over a similar range to that of dingoes (Letnic *et al.* 2009). Why Allen *et al.* recorded such low fox activity is puzzling. One explanation could be that availability of prey for foxes was low at the times and in the places where Allen *et al*. worked, so foxes were unable to increase when released from the effects of baiting; alternatively, dingo activity might have remained sufficiently high to prevent foxes (a possibility also raised by Allen *et al*.) from increasing in unbaited areas. Supporting this latter interpretation, Letnic *et al.*’s (2009) surveys found that fox activity was greatest at levels of dingo activity lower than those recorded at any of the sites sampled by Allen *et al.*

For feral cats and goannas, a direct impact of baiting on activity would be more surprising. Feral cats are much less likely than foxes to take meat baits, because they have a strong preference for hunting of live prey [9,10]. Goannas could well consume baits, but reptiles have high tolerance to the poison (Compound 1080) that is used in programs aimed at control of dingoes and foxes in Australia [11]. A further problem in claiming responses to baiting by mesopredators, including feral cats and dingoes, is that the monitoring technique used by Allen *et al*. – sand-plot tracking – is relatively insensitive to changes in activity of cats and goannas [12].

## Conclusion

The question this study cannot answer is: what would have happened if the poisoning programs implemented by Allen *et al.* had produced large and sustained reductions in dingo activity? This question is an important one, because while baiting seems to have had little effect in this study, there is evidence that it can be effective in reducing dingo activity [1,5]. Other research suggests that the effects of reduced dingo activity could have included increased abundance or activity of foxes and cats [5,13] or, if baiting exerted control over foxes as well as dingoes, even stronger increase in cats as a result of the suppression of the two larger predators [14,15]. In addition, it is important to highlight that Allen *et al.*’s results are not relevant to areas where broad-scale aerial baiting is employed, or where ground baiting is coordinated over large areas, or conducted in conjunction with exclusion fencing. These measures are likely to increase the efficacy of baiting in reducing dingo populations.

The management of dingoes is a highly conflicted and frequently emotional issue in rural Australia. There is an urgent need to find approaches that can balance the needs of agricultural production with environmental conservation. Achieving this balance requires rigorous evaluation of evidence on the effectiveness of alternative strategies of dingo management and their environmental consequences. We commend Allen *et al.* for taking an experimental approach to this problem. Unfortunately, they have not conducted a rigorous analysis of their data. Especially, we find little or no support for their inference that the poison baiting programs reported in their paper had any effect on activity of wild predators, contrary to their view that baiting suppressed activity of all medium-sized and large vertebrate predators. We conclude that their study does not provide useful evidence on the environmental costs of lethal control of dingoes.

## Competing interests

The authors declare that they have no competing interests.

## Authors’ contributions

All authors contributed equally to the content of the paper; CNJ led the writing. All authors read and approved the final manuscript.

## Authors’ information

CNJ is Professor of Wildlife Conservation at the School of Zoology, University of Tasmania; MSC is Senior Lecturer at the School of Biological Sciences, University of Sydney; CRD is Professor in Terrestrial Ecology at the School of Biological Sciences, University of Sydney; MIL is ARC Future Fellow at the Centre for Ecosystem Science, School of Biological Earth and Environmental Sciences, University of New South Wales; TMN is Postdoctoral Scholar at the Department of Forest Ecosystems and Society, Oregon State University; DGN is Postdoctoral Scholar at the Centre for Integrative Ecology and School of Life and Environmental Sciences, Deakin University; EGR is Senior Lecturer at the Centre for Integrative Ecology and School of Life and Environmental Sciences, Deakin University; ADW is Adjunct Lecturer at the School of Marine and Tropical Biology, James Cook University.
